# RNA-sequencing reveals positional memory of multipotent mesenchymal stromal cells from oral and maxillofacial tissue transcriptomes

**DOI:** 10.1186/s12864-020-06825-2

**Published:** 2020-06-22

**Authors:** Satoru Onizuka, Yasuharu Yamazaki, Sung-Joon Park, Takayuki Sugimoto, Yumiko Sone, Sebastian Sjöqvist, Michihiko Usui, Akira Takeda, Kenta Nakai, Keisuke Nakashima, Takanori Iwata

**Affiliations:** 1 Division of Periodontology, Department of Oral Function, Kyushu Dental University, 2-6-1, Manazuru, Kokurakita-ku, Kitakyushu City, Fukuoka, 803-8580 Japan; 2 Department of Plastic and Aesthetic Surgery, Kitasato University School of Medicine, 1-15-1 Kitasato, Minami, Sagamihara, Kanagawa 252-0375 Japan; 3 Human Genome Center, The Institute of Medical Science, The University of Tokyo, 4-6-1 Shirokanedai, Minato-ku, Tokyo, 108-8639 Japan; 4 Institute of Advanced Biomedical Engineering and Science, Tokyo Women’s Medical University, 8-1 Kawada-cho, Shinjuku-ku, Tokyo, 162-8666 Japan; 5 Department of Periodontology, Graduate School of Medical and Dental Sciences, Tokyo Medical and Dental University, 1-5-45 Yushima, Bunkyo-ku, Tokyo, 113-8510 Japan

**Keywords:** Multipotent mesenchymal stromal cells, RNA-sequencing, HOX genes, Maxillofacial bone, Iliac bone, Periodontal ligament

## Abstract

**Background:**

Multipotent mesenchymal stromal cells (MSCs) can be isolated from numerous tissues and are attractive candidates for therapeutic clinical applications due to their immunomodulatory and pro-regenerative capacity. Although the minimum criteria for defining MSCs have been defined, their characteristics are known to vary depending on their tissue of origin.

**Results:**

We isolated and characterized human MSCs from three different bones (ilium (I-MSCs), maxilla (Mx-MSCs) and mandible (Md-MSCs)) and proceeded with next generation RNA-sequencing. Furthermore, to investigate the gene expression profiles among other cell types, we obtained RNA-seq data of human embryonic stem cells (ESCs) and several types of MSCs (periodontal ligament-derived MSCs, bone marrow-derived MSCs, and ESCs-derived MSCs) from the Sequence Reads Archive and analyzed the transcriptome profile. We found that MSCs derived from tissues of the maxillofacial region, such as the jaw bone and periodontal ligament, were HOX-negative, while those derived from other tissues were HOX-positive. We also identified that *MSX1, LHX8,* and *BARX1*, an essential regulator of craniofacial development, were strongly expressed in maxillofacial tissue-derived MSCs. Although MSCs may be divided into two distinct groups, the cells originated from over the neck or not, on the basis of differences in gene expression profile, the expression patterns of all CD antigen genes were similar among different type of MSCs, except for ESCs.

**Conclusions:**

Our findings suggest that MSCs from different anatomical locations, despite meeting general characterization criteria, have remarkable differences in gene expression and positional memory. Although stromal cells from different anatomical sources are generally categorized as MSCs, their differentiation potential and biological functions vary. We suggested that MSCs may retain an original tissue memory about the developmental process, including gene expression profiles. This could have an important impact when choosing an appropriate cell source for regenerative therapy using MSCs.

## Background

Multipotent mesenchymal stromal cells (MSCs) are capable of clonogenic proliferation and differentiation into all mesodermal lineages [1]. They can be isolated from several tissues, such as bone marrow [2], adipose tissue [3], periosteum [4], and periodontal ligaments [5, 6], and have been used therapeutically for tissue regeneration and autoimmune disease treatment because of their multipotency, immunomodulatory properties, and ability to mediate trophic factors [7]. Researchers have also suggested that cells originating from bone tissue, for instance, femurs, iliac, and alveolar bone, possess MSC-like characteristics [8–10].

In the case of bone grafting, progenitor cells that can differentiate into osteoblasts exist in bone tissue and are used in autologous bone grafting for the reconstruction of bony defects. Although several biomaterials have recently attracted attention for their use in bone regeneration [11], the gold standard of clinical bone repair strategies remains the transplantation of autologous bone grafts. Autogenous iliac bone grafting is usually performed to close the bony defects at the alveolar cleft [12]. The iliac bone is the most common donor site for autologous bone grafts because of the availability of sufficient bone and easy access to cancellous bone [13]. Moreover, since autogenous iliac bone is harvested from the patient, it is considered nonimmunogenic and histocompatible. Autogenous iliac bone grafting is generally applied before eruption of the permanent canines, which offers the advantages of stabilization of the maxillary dental arch, creation of bony support for teeth adjacent to the alveolar cleft, closure of the oronasal fistulas, and enhancement of orthodontic and prosthetic treatment [14, 15].

However, the harvesting of autogenous bone grafts is associated with risks of donor site morbidity, such as postoperative pain, altered sensation, donor site infection, and scarring [16]. To avoid this, new strategies for bone regenerative therapy have been investigated, including bioresorbable scaffolds, growth factors such as bone morphogenetic proteins and fibroblast growth factor, and gene therapy [17–20]. Additionally, stem cell transplantation is a promising alternative to autologous bone grafting, and a combination of cultured MSCs and biomaterials was shown to be effective in various animal models for repairing bony defects [21–23]. However, it remains unclear which source of MSCs is the most suitable for bone regeneration.

The maxillofacial skeleton is derived from the cranial neural crest whereas the remainder of the skeleton originates from the mesoderm [24, 25]. It is therefore fundamental to determine whether progenitor cells originating from bone tissue derived from mesoderm can completely regenerate or repair maxillofacial bone. Although MSCs derived from various tissue satisfy the international minimal defining criteria, these cells exhibit different characteristics in terms of proliferation ability, differentiation potential, and gene expression profiles [26–28]. Therefore, to investigate variations in differentiation abilities and gene expression patterns among various types of MSCs, we performed differentiation assay and RNA-sequencing (RNA-seq) using next-generation sequencing analysis. Our results suggest that MSCs derived from different types of tissue, especially the cells originated from over the neck or not, have positional memories and varying gene expression profiles that may influence their cellular characteristics.

## Results

### MSCs from different anatomical locations exhibit varying differentiation potential

**Fig. 1:**
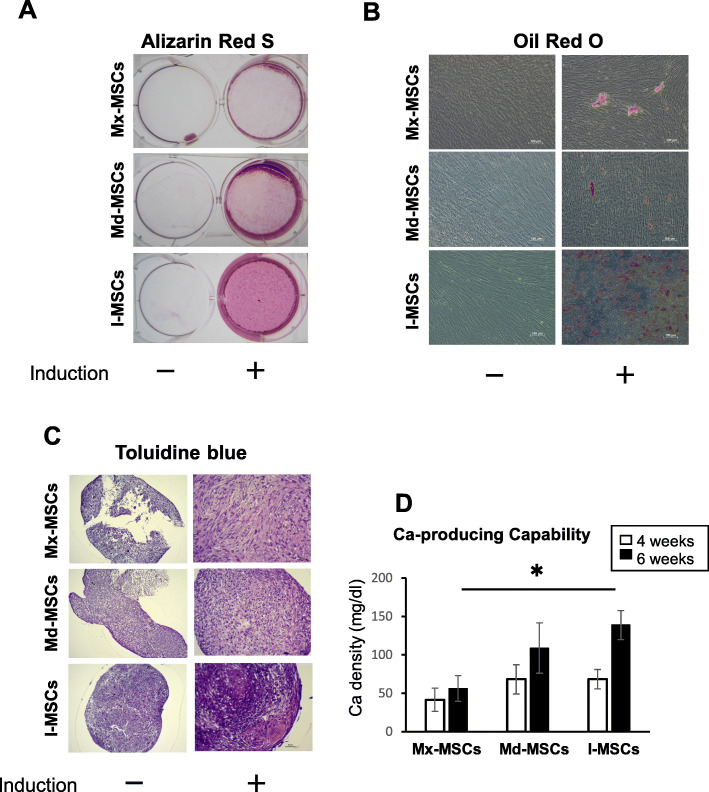
Differentiation potential of each type of bone-derived MSCs. **a** Alizarin red S staining of MSCs cultured in ODM or complete medium for 6 weeks. –, MSCs cultured in complete medium for 6 weeks; +, MSCs cultured in ODM for 6 weeks. **b** Oil red O staining of MSCs cultured in ADM or ODM for 6 weeks. –, MSCs cultured in ODM for 6 weeks; +, MSCs cultured in ODM for 3 weeks, then in ADM for 3 weeks. c Toluidine blue staining of MSCs cultured in CDM or complete medium for 3 weeks. –, MSCs cultured in complete medium for 3 weeks; +, MSCs cultured in ODM for 3 weeks. These images show the representative data from three different samples; a similar tendency was observed in other MSCs. d Calcium was produced when MSCs were cultured in ODM for 4 and 6 weeks. Bars represent the mean ± S.D. of triplicate samples. Asterisks denote significant difference (*p* <  0.05, Tukey’s HSD)

First, to verify that the cells used in this study have multipotency to differentiate into several cell types, we performed differentiation assays (Fig. 1 and Figure S1). Alizarin red S staining showed that all types of MSCs could differentiate into osteoblasts and form mineralized nodules, but the degree of mineralization was lower for maxilla-derived MSCs (Mx-MSCs) than for other MSCs (Fig. 1a). Oil Red O and toluidine blue staining assay showed that ilium-derived MSCs (I-MSCs) had the greatest potential for adipogenic and chondrogenic differentiation compared with Mx-MSCs and mandible-derived MSCs (Md-MSCs) (Fig. 1b and c). In the calcium content assay, I-MSCs and Md-MSCs produced more Ca than Mx-MSCs when the cells were cultured in ODM for 4 weeks (Fig. 1d). Moreover, Ca levels in I-MSCs were significantly higher than in Mx-MSCs when the cells were cultured in ODM for 6 weeks (Fig. 1d).

### The general profile of RNA-seq data

**Fig. 2:**
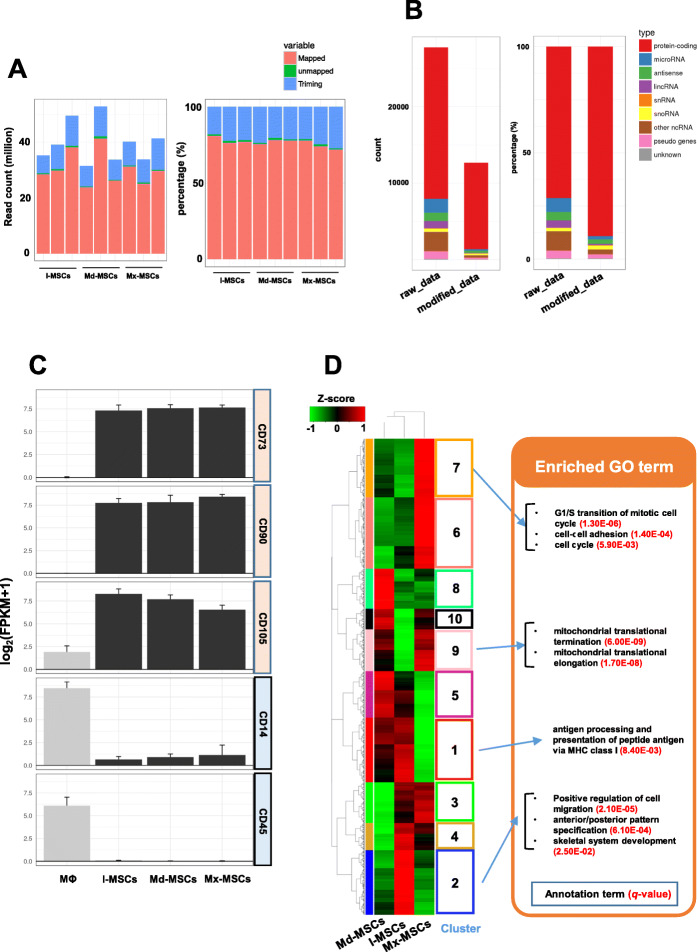
Gene expression profile and GO term enrichment analysis of bone-derived MSCs. **a** Cumulative number (left) and ratio (right) of single-ended reads generated from the Ion Proton system (1e+ 07 = 10,000,000). **b** Cumulative number (left) and ratio (right) of sequencing reads aligned to the human reference genome (hg19). Each color represents a different RNA biotype, such as protein-coding RNA, non-coding RNA, and unknown. ‘modified_data’ is Refseq genes expressed in at least one group with FPKM ≥1. **c** The expression levels of CD14, CD45, CD73, CD90, and CD105 among each type of cells. The RNA-seq data (accession number: SRA245718) of human Macrophage (MΦ) were used as control. d Heatmap showing the relative expression profile of 12,676 genes (FPKM ≥1) among I-MSCs, Mx-MSCs, and Md-MSCs. Scaled expression values are color-coded according to the legend on the left. These genes were categorized into 10 patterns by a hierarchical clustering approach. The genes in each pattern were over-represented by specific GO biological process terms (*q* <  0.05)

The Ion Proton system generated 31.5–52.8 million single-ended 50–200 bp reads from nine samples (Fig. 2a). Unmapped reads were reduced to less than 5% by removing low-quality reads. Genes with low expression (fragments per kilobase of exon per million mapped fragments (FPKM) < 1.0) were removed from the three groups, leaving a total of 12,676 genes. Most non-coding RNAs (long intergenic noncoding RNAs, microRNAs, small nuclear RNAs, small nucleolar RNAs, and pseudogenes) had a FPKM < 1 in all samples (Fig. 2b). To verify that the cells used in this study have specific MSC markers, we analyzed the gene expression profile of CD14, CD45 (PTPRC), CD73 (NT5E), CD90 (THY1), and CD105 (ENG). RNA-seq data of human macrophage (MΦ) used as control ware obtained from the Sequence Reads Archive (DDBJ accession number: SRA245718). In three types of MSCs, the expression levels of CD73, CD90, and CD105, widely known as specific surface markers of MSCs, were higher than those in MΦ. On the other hand, the three types of MSCs had little expression of CD14 and 45 (Fig. 2c). Using genes expressed in at least one of the groups with FPKM ≥1, we detected 10 different gene clusters in MSCs that exhibited distinct expression patterns (Fig. 2d). The genes in each cluster were significantly enriched by specific GO terms. For instance, GO terms associated with the cell cycle and cell adhesion detected from cluster 7 consisted of genes up-regulated in Mx-MSCs compared with other MSCs, while GO terms associated with cell migration detected from cluster 2 consisted of genes up-regulated in I-MSCs compared with other MSCs. These results suggested that the cell growth and migration properties of each cell type were different.

### GO term enrichment analysis reveals differences between I-MSCs and Mx−/Md-MSCs

**Fig. 3:**
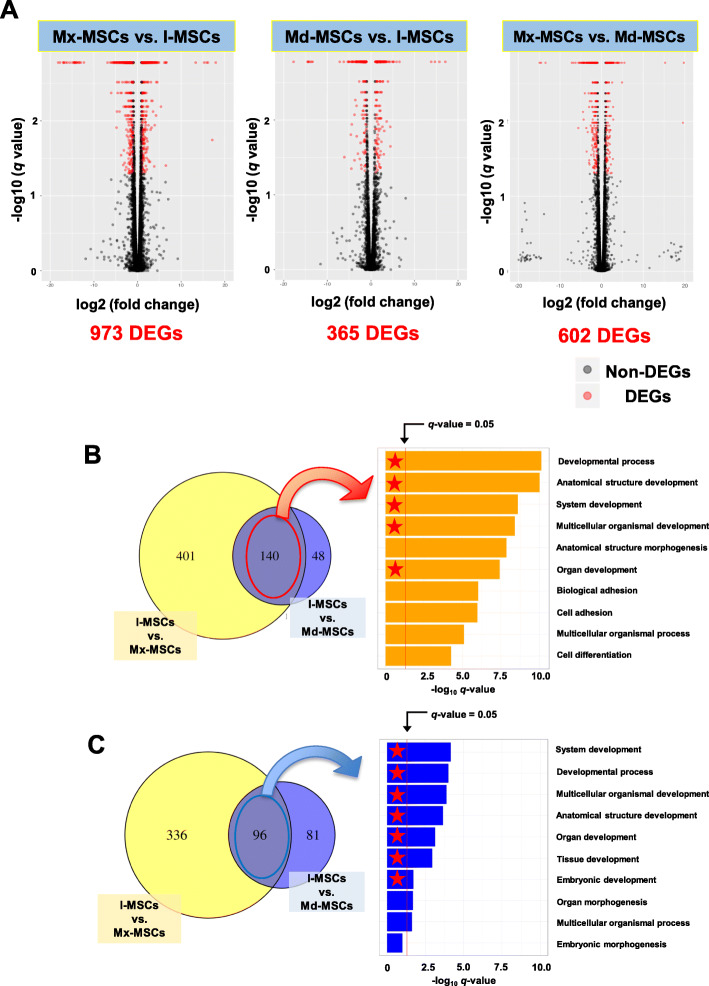
Profile of specific differentially expressed genes (DEGs) and GO term enrichment analysis using the DEGs. **a** Volcano plot showing all profiled genes. Gray and red circles represent non-DEGs and DEGs, respectively. Statistical analysis detected DEGs between two samples using a threshold (*q*-value < 0.05 and a more than two-fold change in FPKM). **b**, **c** High (b) and low (c) expressed DEGs in I-MSCs compared with Mx / Md-MSCs. Venn diagram shows the number of DEGs in I-MSCs vs. Mx-MSCs (yellow circle) and I-MSCs vs. Md-MSCs (blue circle). Bar plot shows the top 10 enriched GO terms identified from co-DEGs (mid area in the Venn diagram) using statistical analysis (horizontal axis represents *–log*_10_[*q*-value]). Red stars indicate the GO term involved in development

To detect potential key regulators of each sample, we investigated DEGs between Mx-MSCs vs. I-MSCs, Md-MSCs vs. I-MSCs, and Mx-MSCs vs. Md-MSCs, and identified 973, 365, and 602 DEGs, respectively (Fig. 3a). To investigate differences in skeletal development and endochondral and intramembranous ossification, GO term enrichment analysis for I-MSC-specific DEGs was performed. A total of 140 DEGs were up-regulated in I-MSCs compared with Mx- and Md-MSCs (U-DEGs) (Fig. 3b). DAVID annotation of these DEGs revealed that most of the top 10 enriched GO terms were involved in development. A total of 96 DEGs were down-regulated in I-MSCs compared with Mx- and Md-MSCs (D-DEGs), and most of the top 10 enriched GO terms were also involved in development (Fig. 3c). These results indicate that I-MSCs and Mx−/Md-MSCs are regulated by genes involved in development.

### Whole transcriptome analysis shows that MSCs derived from tissue in the maxillofacial region are HOX-negative

Based on GO term analysis, significant DEGs between I-MSCs and MSCs derived from jaw bone (Mx−/Md-MSCs) were selected in order of the largest fold change. The top 20 up-regulated DEGs in I-MSCs compared with Mx- or Md-MSCs revealed that most genes specifically expressed in I-MSCs were from the HOX gene family although *WISP3* was also specifically expressed in I-MSCs (Tables 1 and 2).

**Table 1:** The top 20 up-regulated DEGs in I-MSCs compared with Mx-MSCs

	FPKM (Mean)		FC (log2)
	I-MSCs	Mx-MSCs.	
HOXC6 ^a^	30.353	< 0.0001	18.211
HOXC10 ^a^	22.068	< 0.0001	17.752
HOXC8 ^a^	12.962	< 0.0001	16.984
HOXB4 ^a^	12.954	< 0.0001	16.983
HOXC9 ^a^	12.075	< 0.0001	16.882
HOXA3 ^a^	1.255	< 0.0001	16.487
HOXA11 ^a^	2.189	< 0.0001	14.912
HOXD4 ^a^	2.538	< 0.0001	14.631
HOXA5 ^a^	3.083	< 0.0001	14.528
HOXA7 ^a^	7.18	< 0.0001	14.347
HOXA4 ^a^	2.084	< 0.0001	14.345
HOXC-AS1 ^a,b^	2.071	< 0.0001	14.338
HOXC-AS2 ^a,b^	1.64	< 0.0001	14.001
HOXC11 ^a^	1.627	< 0.0001	13.99
HLA-DQB1	0.81	< 0.0001	13.982
HOXA6 ^a^	4.767	< 0.0001	13.616
HOXA-AS2 ^a,b^	2.081	< 0.0001	13.366
WISP3	1.056	< 0.0001	13.331
HOXA1 ^a^	2.363	< 0.0001	12.984
HOTAIRM1 ^a,b^	9.188	< 0.0001	12.278

^a^HOX gene family, ^b^non-coding RNAs, FC: Fold change of FPKM value between two samples (log2 of FC)

**Table 2:** The top 20 up-regulated DEGs in I-MSCs compared with Md-MSCs

	FPKM (Mean)		FC (log2)
	I-MSCs	Md-MSCs.	
VTRNA1–1 ^b^	336.731	< 0.0001	21.693
HOXC10 ^a^	22.068	< 0.0001	17.752
HOXA1 ^a^	2.363	< 0.0001	14.528
HOXA4 ^a^	2.084	< 0.0001	14.347
HOXA-AS2 ^a,b^	2.081	< 0.0001	14.345
HOXC-AS2 ^a,b^	1.64	< 0.0001	14.001
HOXA3 ^a^	1.255	< 0.0001	13.616
WISP3	1.056	< 0.0001	13.366
HOXC6 ^a^	30.353	0.214447	7.145
CACNG8	10.729	0.145148	6.208
HOXC9 ^a^	12.075	0.179618	6.071
LOC400043 ^b^	11.018	0.28332	5.281
CELSR3	7.617	0.223098	5.093
HOXB7 ^a^	16.183	0.494379	5.033
CHI3L1	18.989	0.660538	4.845
ROR2	1.729	0.0724027	4.577
HIST1H2BH	13.291	0.606649	4.454
UNC5C	1.071	0.0503351	4.412
TENM2	1.548	0.0745513	4.376
HOXA11 ^a^	2.189	0.106172	4.366

^a^HOX gene family, ^b^non-coding RNAs, FC: Fold change of FPKM value between two samples (log2 of FC)

**Fig. 4:**
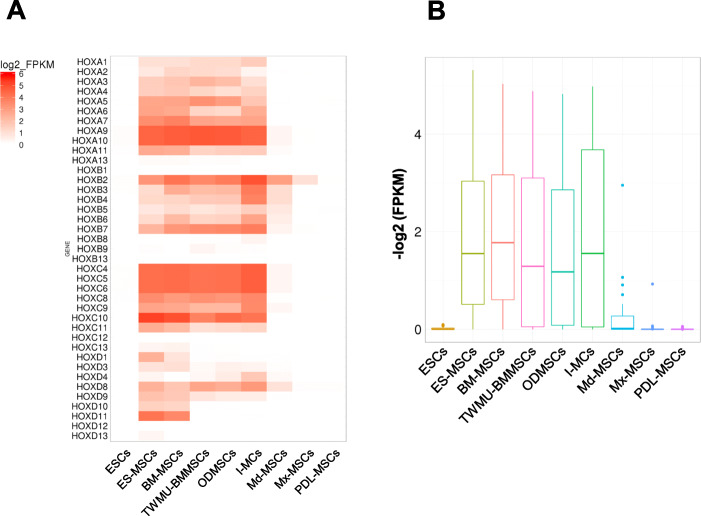
Gene expression profile of all HOX genes among MSCs and ESCs. **a** Heat map showing the degree of expression levels (*log*2 of FPKM value) of all HOX genes. **b** Box plot showing the distribution of expression levels (*log*2 of FPKM value) of HOX genes (except HOX genes with FPKM = 0 in all samples). ESCs, human embryonic stem cells; ES-MSCs, human ESC-derived MSCs; BM-MSCs, human bone marrow-derived MSCs; TWMU-BMMSCs, BM-MSCs owned by TWMU; ODMSCs, TWMU-BMMSCs cultured with ODM; PDL-MSCs, periodontal ligament-derived MSCs

Next, we further evaluated the expression profile of all HOX genes among the three samples. We obtained HOX FPKM values, and analyzed the degree and distribution of HOX gene expression levels (Figures S2A and S2B). I-MSCs were found to have a HOX-positive profile, while MSCs derived from jaw bones (Mx−/Md-MSCs) had HOX-negative profiles. To investigate the gene expression profiles of other cell types, we obtained RNA-seq data from human embryonic stem cells (ESCs), human ESC-derived MSCs (ES-MSCs), and human bone marrow-derived MSCs (BM-MSCs) from the Sequence Reads Archive (DDBJ accession number: SRA245478) and previous RNA-seq data owned by Tokyo Women’s Medical University (TWMU) from human periodontal ligament-derived MSCs (PDL-MSCs), BM-MSCs (TWMU-BMMSCs), and BM-MSCs cultured in ODM (ODMSCs). Figure 4a and b show that ECSs and PDL-MSCs also possessed a HOX-negative profile, similar to Mx- and Md-MSCs. Interestingly, although ESCs had a HOX-negative profile, the expression patterns of HOX genes changed to HOX-positive after the cells differentiated into MSCs. There was less change in the expression profile of HOX genes between TWMU-BMMSCs and ODMSCs, suggesting that HOX mRNA expression may not be affected by ODM.

### Characteristics of gene expression patterns in different types of tissue-derived MSCs and ESCs

To investigate whether HOX-negative MSCs such as PDL-MSCs, Mx-MSCs, and Md-MSCs showed similar gene expression patterns, U-DEG and D-DEG mRNA expression was analyzed among nine samples. U-DEG and D-DEG expression patterns in PDL-MSCs were similar to those in Mx−/Md-MSCs, while U-DEG and D-DEG expression patterns in MSCs derived from bone marrow (BM-MSCs, TWMU-BMMSCs, and ODMSCs) and ES-MSCs were similar to those in I-MSCs (Figures S3A and S3B). These results revealed the similarity of HOX-negative MSCs and the similarity of HOX-positive MSCs with respect to gene expression profiles in U-DEGs and D-DEGs.

**Fig. 5:**
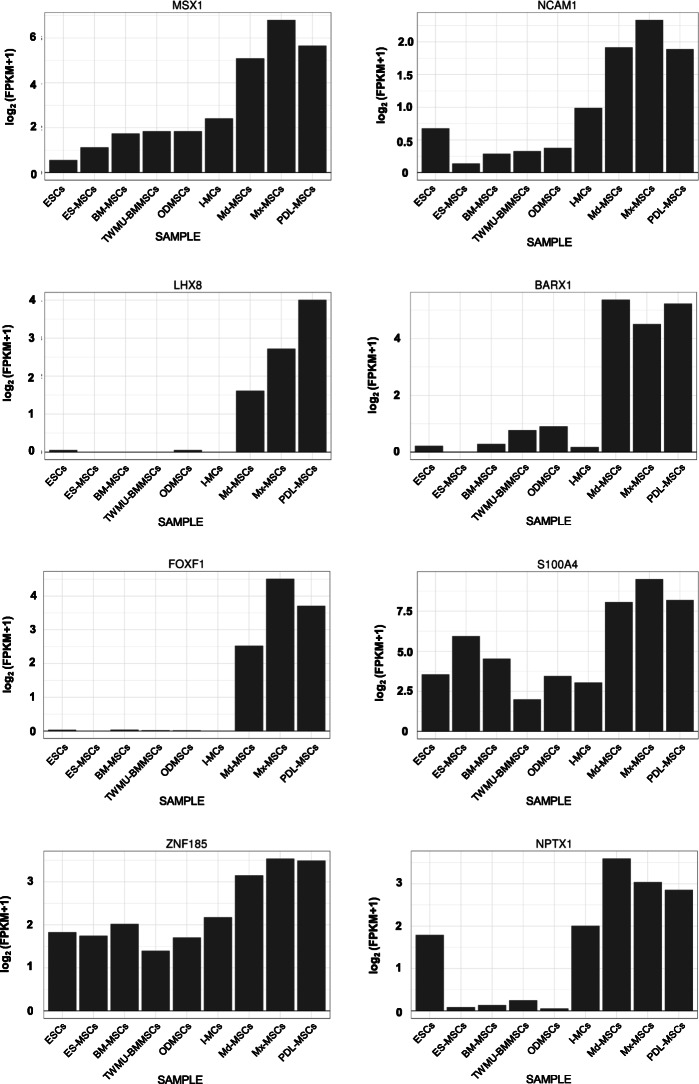
Specific up-regulated DEGs in oral and maxillofacial tissue-derived MSCs. The vertical axis represents gene expression levels with *log*2 of [FPKM + 1]

Next, we investigated the specific up-regulated DEGs in maxillofacial region-derived MSCs (Mx-MSCs, Md-MSCs, and PDL-MSCs; Mfr-MSCs) compared with the others. We identified 8 genes with FPKM levels in each Mfr-MSC sample that were twice as high as in the other six samples: *MSX1*, *NCAM1*, *LHX8*, *BARX1*, *FOXF1*, *S100A4*, *ZNF185*, and *NPTX1* (Fig. 5). The expression levels of *LHX8*, *BARX1*, *FOXF1*, and *NCAM1* were quite low (FPKM < 1.0) in non Mfr-MSCs, indicating that they could be used as specific marker genes for Mfr-MSCs.

### Gene expression profiles of CD antigens in each sample

**Fig. 6:**
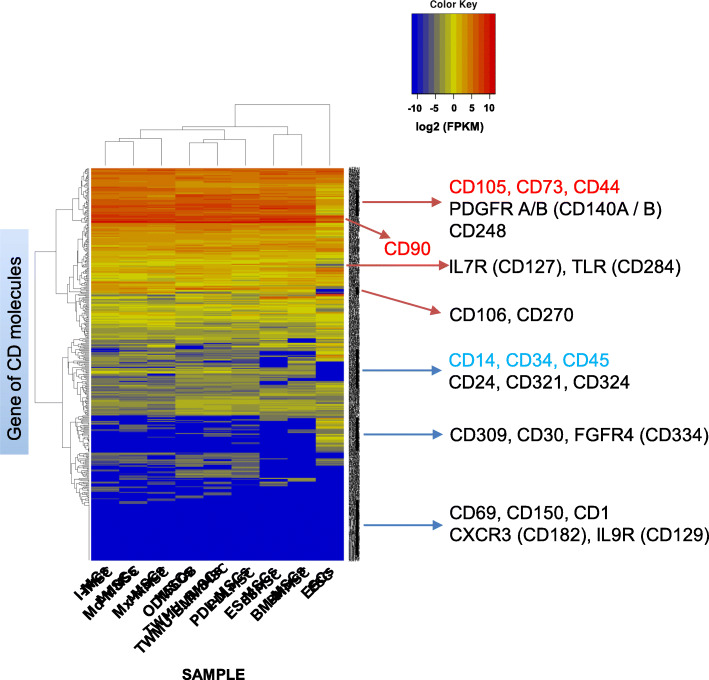
Gene expression profile of CD molecules in each sample. Heatmap showing the degree of expression levels (*log*2 of FPKM value) of all CD genes. Scaled expression values are color-coded according to the legend on the left. Genes are hierarchically clustered by the similarity of their expression profiles over the set of samples, and the samples are hierarchically clustered by the similarity of expression patterns over their expression profile. The described genes (right side of heatmap) are representative CD molecules. Red letters represent positive markers for MSCs, and blue letters represent negative markers for MSCs

Fig. 6 shows the gene expression profile of CD molecules in each type of MSC and ESC. The mRNA expression of positive markers for MSCs, such as CD105, CD73, and CD44, was significantly higher (FPKM > 50) in all MSCs compared with ESCs (FPKM < 3.0). CD90, an MSC-positive marker, was highly expressed (FPKM > 100) in both MSCs and ESCs. The mRNA expression of MSC-negative markers, such as CD14, CD34, and CD45, showed low levels (FPKM < 2.0) in MSCs and ESCs. CD106 and CD270 were not expressed (FPKM = 0) in ESCs, although these genes demonstrated high or low expression in MSCs. The expression patterns of all CD antigen genes were similar among different type of MSCs, except for ESCs.

## Discussion

Although several studies have suggested that MSCs can be isolated from various tissues, such cells have different in vitro characteristics such as proliferation abilities, differentiation potentials, and gene expression profiles [26, 27]. In the present study, I-MSCs readily differentiated into both osteoblasts and adipocytes; in contrast, Mx-MSCs and Md-MSCs, derived from bone of the maxillofacial region, hardly differentiated into adipocytes, and the osteogenic potential of Mx-MSCs was significantly lower than that of I-MSCs.

In support of this, alveolar BM-MSCs were previously found to show little differentiation into adipocytes and chondrocytes, in contrast to ilium BM-MSCs [29]. However, the differentiation potential and HOX genes profile of maxilla differ from those of the mandible, which may reflect the fact that Md-MSCs were isolated from bone tissue of the mandibular angle. Although the maxillofacial bone is usually formed by intramembranous ossification [30, 31], the condyle, coronoid, and angular of the mandible develop through endochondral ossification [32]. Therefore, cells in the mandibular angle have different characteristics to those in the maxilla even though they are both maxillofacial bone. RNA-seq findings of our study also showed a difference in gene expression profiles between Mx-MSCs and Md-MSCs, which should be studied further.

We focused our attention on gene expression profile among MSCs derived from maxillofacial bone of neural crest origin and iliac bone of mesoderm origin in RNA-seq data analysis. While these cells generally are classified as MSCs, the gene expression profiles, particularly the HOX gene family are greatly different. MSCs originating from the ilium were shown to be HOX-positive, while those originating from the maxillofacial bone were HOX-negative. This study had a limitation in that distribution of donor age among each type of MSCs was uneven. Since primary culture was performed using remaining bone tissue obtained from bone grafting in cleft lip and palate patients or osteotomy in jaw deformity patients, donor age was dependent on operation timing. However, our previous study showed that ALP activity of I-MSCs cultured in osteoinductive medium for 1 week was not different from that of Md-MSCs [33]. Therefore, it is quite likely that HOX genes profile of MSCs is not related to donor age. Furthermore, PDL-MSCs residing in the maxillofacial region were also HOX-negative. Interestingly, some researchers have suggested that human fibroblasts retain the memory of the embryonic HOX status both in vitro and in vivo [34, 35]. Therefore, MSCs may retain memory from the developmental process, whether the cells derived from neural crest or not.

Our findings are consistent with previous studies, which showed that the Hox status of tibiae was positive, while that of the mandibular was negative [28, 36]. Moreover, Matsubara et al. showed that alveolar bone marrow-derived MSCs hardly differentiated into chondrocytes compared with iliac bone marrow-derived MSCs [29], while Iwata et al. found that the chondrogenic differentiation capacity of PDL-MSCs was lower than that of adipose-derived or bone marrow-derived MSCs [37]. Thus, HOX-negative MSCs might be less prone to differentiate into chondrocytes compared with HOX-positive MSCs. Because the progenitor of HOX-negative maxillofacial bone typically forms bone via intramembranous ossification and does not follow the differentiation process into chondrocytes, it is assumed that MSCs originating from the same region may be programmed not to differentiate into chondrocytes. However, our RNA-seq data showed that several HOX genes were expressed in Md-MSCs, which therefore may not be completely HOX-negative despite the fact that the cells originated from maxillofacial bone. The cause of this discrepancy may reflect the characteristics of Md-MSCs isolated from the mandibular angle which develop through endochondral ossification. The results also suggest that the HOX gene cluster regulates the chondrogenic differentiation of MSCs.

Our RNA-seq data revealed that Mfr-MSCs were HOX-negative, and identified specifically up-regulated genes in Mfr-MSCs, including *MSX1*, *NCAM1*, *LHX8*, *BARX1*, *FOXF1*, *S100A4*, *ZNF185*, and *NPTX1*. A previous study identified severe craniofacial defects in *Msx1*^*−/−*^ mice including the failure of palatal shelves to elevate and fuse, mandible and middle ear ossicle deformities, the absence of molars, and delayed ossification [38, 39]. Other research indicated that mutations in human *MSX1* are associated with cleft palate and tooth agenesis [40, 41]. Additionally, a targeted *Lhx8* mutation in mice caused cleft palate in around 60% of animals [42]. Moreover, the microdeletion of *BARX1* in humans is associated with craniofacial developmental disorders such as microstomia and mandibular retrusion [43], while BARX1 was recently shown to function as a direct downstream factor of GATA4 in neural crest development [44]. Hence, the Mfr-MSC-specific up-regulated genes obtained from our RNA-seq data may serve as key factors for neural crest or craniofacial bone (intramembranous ossification) development. In contrast, previous studies found that the ectopic expression of several Hox genes induced craniofacial and skeletal malformation in transgenic mice [45, 46]. Therefore, some Hox genes appear not to be expressed in the cranial region during development. These findings suggest that the maxillofacial region is regulated by different transcription factors to other regions during development, and it is inferred from our RNA-seq data that MSCs inherit site-specific memories.

The International Society for Cellular Therapy (ISCT) proposed sets of minimum criteria to identify MSCs [47]. In fact, our RNA-seq data revealed similar expression patterns of specific cell surface marker genes among each type of MSCs, except for ESCs. Moreover, despite differences in the differentiation capacity among MSC types, these cells nevertheless possessed a multilineage differentiation ability. Therefore, although cells that fulfill the minimum ISCT criteria are categorized as the same MSCs, it may be more effective to use MSCs derived from the same tissue for cell therapy considering that they inherit the characteristics of the tissue of origin. Indeed, adipose-derived MSCs possessed a high potential for adipogenesis [48], while cartilage-derived MSCs had a high potential for chondrogenesis in an in vitro assay [49]. In in vivo studies, PDL-MSCs showed the greatest potential to regenerate periodontal ligament tissue compared with other sources of MSCs [50]. Considering the varied characteristics of MSCs, it is important to carefully consider the most suitable source for the regeneration therapy of target tissue.

## Conclusions

In this study, Mx / Md-MSCs were shown to have a different gene expression profile compared to I-MSCs, based on RNA-sequencing. In particular, since HOX status of MSCs originating from oral and maxillofacial tissues was HOX-negative, the expression patterns of HOX genes are quite different whether the cells derived from over the neck or not. Although stromal cells from different anatomical sources are generally categorized as MSCs, their differentiation potential and biological functions vary. Our findings suggest that MSCs may retain an original tissue memory about the developmental process, including gene expression profiles. This could have an important impact when choosing an appropriate cell source for regenerative therapy using MSCs.

## Methods

### Specimens and cell cultures

Nine bone specimens (ilium: three cases, maxilla: three cases, mandible: three cases; Table S1) were harvested from conventional surgery conducted by the Department of Plastic and Aesthetic Surgery at Kitasato University. Table S1 shows patient profiles, including sex, age, and type of bone. Small pieces of bone were cultured in complete medium containing 10% fetal bovine serum (MP Biomedicals LLC, Santa Ana, CA) at 37 °C with 95% humidity and 5% CO_2_. The complete medium consisted of alpha-minimum essential medium (Thermo Fisher Scientific, Waltham, MA) with 100 U/mL penicillin, 100 μg/mL streptomycin (Thermo Fisher Scientific), and 1 ng/mL basic fibroblast growth factor (R&D Systems, Minneapolis, MN). The medium was changed twice a week during expansion culture. All experiments were conducted with MSCs from second and third passages in this study.

### Differentiation for osteogenesis and adipogenesis

MSCs were cultured in complete medium at confluence, and then seeded in 6-well plates at a density of 1 × 10^5^ cells/well. For osteogenic differentiation, the cells were induced by culturing in osteogenic differentiation medium (ODM), consisting of complete medium supplemented with 50 μM ascorbic acid (Wako Pure Chemical Industries, Osaka, Japan), 10 mM β-glycerophosphate (Calbiochem, San Diego, CA), and 100 nM dexamethasone (Sigma-Aldrich, St Louis, MO). The medium was replaced twice a week.

For adipogenic differentiation, the cells were induced by culturing in adipogenic differentiation medium (ADM), consisting of complete medium supplemented with 10 μg/ml insulin (Wako Pure Chemical Industries), 200 μM indomethacin (Wako Pure Chemical Industries), 500 μM isobutylmethylxanthine (Sigma-Aldrich), and 1 μM dexamethasone. The medium was replaced twice a week.

Chondrogenic differentiation was performed according to the pellet culture method [51]. We used chondrogenic differentiation medium (CDM) consisting of High-glucose DMEM (Gibco) supplemented with 2 mM L-glutamine (MP Biomedicals), 50 μg/mL ascorbic acid-2-phosphate, 100 nM dexamethasone, 100 μg/mL sodium pyruvate, 10 ng/mL human recombinant TGF-β1 (Sigma-Aldrich), and 50 mg/mL ITS+Premix (Corning). The medium was replaced twice a week.

### Examination and evaluation of MSC characteristics

For osteogenic differentiation, MSCs were cultured in ODM at 37 °C for 6 weeks. The ODM was replaced twice a week. For adipogenic differentiation, MSCs were cultured in ODM at 37 °C for 3 weeks. After culturing for 3 weeks, the medium was replaced to ADM, and MSCs were cultured for an additional 3 weeks. As a control, MSCs were continuously cultured in ODM for 6 weeks.

For the calcium (Ca) production assay, MSCs were seeded in 6-well plates at a density of 1 × 10^5^ cells/well and cultured in ODM or complete medium as negative control at 37 °C. After 4 and 6 weeks, Ca levels were evaluated using ESPA Ca (Nipro, Osaka, Japan).

For Ca staining assay, MSCs were seeded in 6-well plates at a density of 1 × 10^5^ cells/well and cultured in ODM or complete medium at 37 °C. After 6 weeks, MSCs were stained with 1.3% alizarin red S solution for 2 min at room temperature. The cells were washed twice with phosphate-buffered saline (PBS), fixed with 100% ethanol, washed twice with distilled water to remove staining solution, and then the plate was allowed to dry.

For lipid staining with oil red O solution, Oil red O staining was performed after adipogenic differentiation. The cells were washed twice with PBS, fixed with 10% formalin, washed once with distilled water and 60% isopropanol, and then stained by Oil red O solution for 20 min.

The chondrogenic differentiation potency was evaluated after 3 weeks culture in CDM. The cell pellet was washed twice with PBS and fixed with 4% formaldehyde overnight at 4 °C. After paraffin embedding, the sample was cut into 5 μm sections and stained with toluidine blue (Wako Pure Chemical Industries).

### Isolation of total RNA and cDNA library preparation for RNA-sequencing (RNA-seq)

For RNA-seqencing, ilium-derived MSCs (I-MSCs), maxilla-derived MSCs (Mx-MSCs), and mandible-derived MSCs (Md-MSCs) were seeded in 100 mm dishes at a density of 5 × 10^5^ cells/dish and cultured in complete medium for 3 days. Total RNA was isolated using the RiboPure RNA Purification Kit (Thermo Fisher Scientific). When the RIN (RNA integrity number) score was > 9, these RNA were used for RNA-seq. Strand-specific RNA libraries were obtained by the Dynabeads mRNA DIRECT Micro Purification Kit and the Ion Total RNA-Seq Kit v2.0 (Thermo Fisher Scientific). Each of RNA-seq template was prepared using the Ion PI Hi-Q OT2 200 Kit, the Ion OneTouch 2, and Ion OneTouch ES systems (Thermo Fisher Scientific). We performed RNA-seq using Ion Proton sequencer with Ion PI Hi-Q Sequencing 200 Kit (Thermo Fisher Scientific).

### Statistical and bioinformatics analysis

Adaptor sequences of fastq file were removed by Cutadapt (v. 1.10), and low-quality bases of fastq file were trimmed by Trimmomatic (v. 0.35). Sequencing reads were aligned to the human reference genome (hg19) by Bowtie2 (v. 2.2.6) and Tophat2 (v. 2.1.0) software [52]. We have performed Tophat2-Cufflinks (v. 2.2.1) packages pipeline to normalize uniquely mapped reads as fragments per kilobase of exon per million mapped fragments (FPKM) [53]. To detect differentially expressed genes (DEGs) between two groups, we used Cuffdiff that calculates a fold change for every gene, a *p*-value, and the false discovery rate with the Benjamini-Hochberg correction (*q*-value) in the Cufflinks packages. DEGs were defined when the *q*-value < 0.05 and the fold change of FPKM was ≥2.0. The hg19 reference and the Refseq annotation were obtained from the UCSC Genome Browser (https://genome.ucsc.edu/). Enriched Gene Ontology (GO) terms were analyzed by the Database for Annotation, Visualization and Integrated Discovery (DAVID; https://david.ncifcrf.gov/) with the annotation dataset of GO biological process. Specific GO terms were gathered from the NCBI database (ftp://ftp.ncbi.nlm.nih.gov/gene/DATA/). Most of plots and graphs of RNA-seq analysis were visualized by ggplot2 and other R packages. Clustering analysis was performed by the function hclust of R packages. All fastq file have been deposited in the DNA Data Bank of Japan (DDBJ) Sequence Read Archive under accession numbers DRA006607–006615. In the statistical analyses for Ca content assay, all of the values are reported as the mean ± standard deviation (SD). Tukey’s HSD test was performed for comparing differences between multiple groups. Differences were considered significant at *p* <  0.05.

## Supplementary information

**Additional file 1: Figure S1.** Osteogenic and adipogenic differentiation potential of all MSC samples. **Figure S2.** Gene expression profile of all HOX genes in I-MSCs, Mx-MSCs, and Md-MSCs. **Figure S3.** Distinct gene expression patterns between Mfr-MSCs and other cells.

**Additional file 2: Table S1.** The information about the subjects.

## Data Availability

RNA-seq data generated and analyzed in this study have been deposited in the DNA Data Bank of Japan (DDBJ) Sequence Read Archive under accession numbers DRA006607–006615 (https://ddbj.nig.ac.jp/DRASearch/study?acc=DRP005861). The other data for subsequent RNA-seq were obtained from the National Center for Biotechnology Information (NCBI) Sequence Read Archive (SRA) database with the following accession numbers SRP055967 (https://trace.ncbi.nlm.nih.gov/Traces/sra/?study=SRP055967). We used human genome assembly GRCh37 (hg19), downloaded from the UCSC genome browser (http://hgdownload.cse.ucsc.edu/downloads.html#human), and the Refseq annotation files were obtained from NCBI (https://ftp.ncbi.nlm.nih.gov/gene/DATA/). All scripts and data are available from the corresponding author on reasonable requests.

## Abbreviations

MSCs: Multipotent mesenchymal stromal cells
I-MSCs: Ilium-derived MSCs
Mx-MSCs: Maxilla-derived MSCs
Md-MSCs: Mandible-derived MSCs
ODM: Osteogenic differentiation medium
OD-MSCs: BM-MSCs cultured in ODM
PDL-MSCs: Periodontal ligament-derived MSCs
ESCs: Embryonic stem cells
ES-MSCs: ESC-derived MSCs
Mfr-MSCs: Maxillofacial region-derived MSCs
RNA-seq: RNA-sequencing
FPKM: Fragments per kilobase of exon per million mapped fragments
MΦ: Macrophage
U-DEGs: Up-regulated DEGs in I-MSCs compared with Mx- and Md-MSCs
D-DEGs: Down-regulated DEGs in I-MSCs compared with Mx- and Md-MSCs
