# Correction to: RNA-sequencing reveals positional memory of multipotent mesenchymal stromal cells from oral and maxillofacial tissue transcriptomes

**DOI:** 10.1186/s12864-020-06939-7

**Published:** 2020-08-11

**Authors:** Satoru Onizuka, Yasuharu Yamazaki, Sung-Joon Park, Takayuki Sugimoto, Yumiko Sone, Sebastian Sjöqvist, Michihiko Usui, Akira Takeda, Kenta Nakai, Keisuke Nakashima, Takanori Iwata

**Affiliations:** 1 Division of Periodontology, Department of Oral Function, Kyushu Dental University, 2-6-1, Manazuru, Kokurakita-ku, Kitakyushu City, Fukuoka 803-8580 Japan; 2 Department of Plastic and Aesthetic Surgery, Kitasato University School of Medicine, 1-15-1 Kitasato, Minami, Sagamihara, Kanagawa 252-0375 Japan; 3 Human Genome Center, The Institute of Medical Science, The University of Tokyo, 4-6-1 Shirokanedai, Minato-ku, Tokyo, 108-8639 Japan; 4 Institute of Advanced Biomedical Engineering and Science, Tokyo Women’s Medical University, 8-1 Kawada-cho, Shinjuku-ku, Tokyo, 162-8666 Japan; 5 Department of Periodontology, Graduate School of Medical and Dental Sciences, Tokyo Medical and Dental University, 1-5-45 Yushima, Bunkyo-ku, Tokyo, 113-8510 Japan

**Correction to: BMC Genomics (2020) 21:417**

**https://doi.org/10.1186/s12864-020-06825-2**

Following publication of the original article [1], the authors identified an error in Figs. 4, 5, 6. In these figures the label for I-MSC’s was incorrectly given as I-MCs. The correct figures are given below and the original article has been updated.

**Fig. 4:**
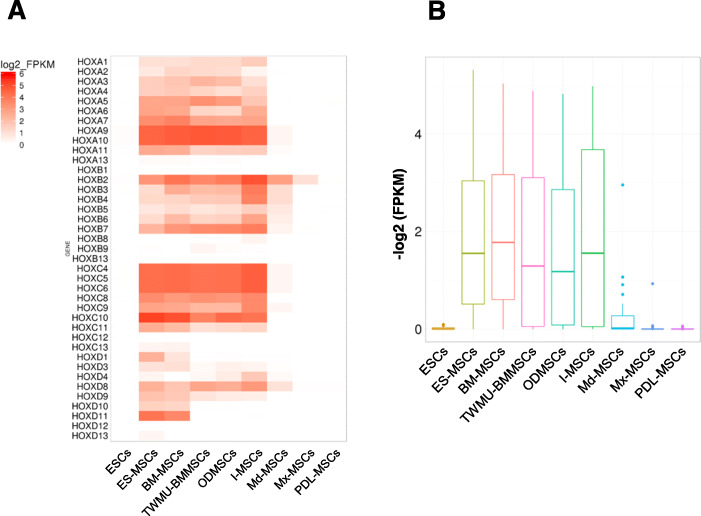
Gene expression profile of all HOX genes among MSCs and ESCs. **a** Heat map showing the degree of expression levels (log2 of FPKM value) of all HOX genes. **b** Box plot showing the distribution of expression levels (log2 of FPKM value) of HOX genes (except HOX genes with FPKM = 0 in all samples). ESCs, human embryonic stem cells; ES-MSCs, human ESC-derived MSCs; BM-MSCs, human bone marrow-derived MSCs; TWMU-BMMSCs, BM-MSCs owned by TWMU; ODMSCs, TWMU-BMMSCs cultured with ODM; PDL-MSCs, periodontal ligament-derived MSCs

**Fig. 5:**
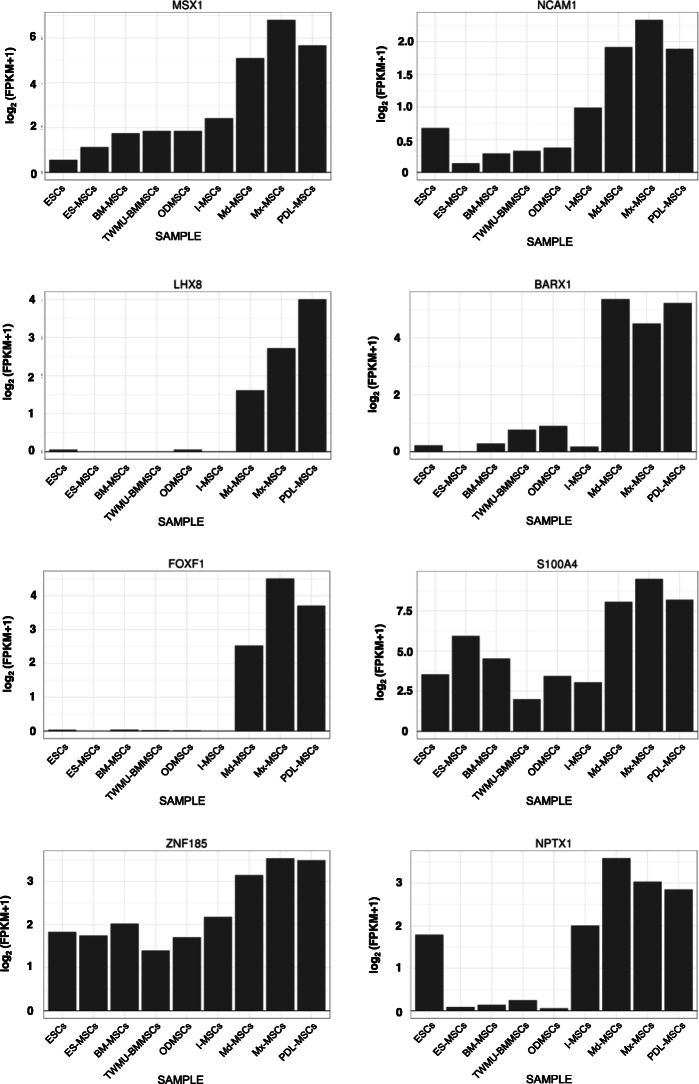
Specific up-regulated DEGs in oral and maxillofacial tissue-derived MSCs. The vertical axis represents gene expression levels with log2 of [FPKM + 1]

**Fig. 6:**
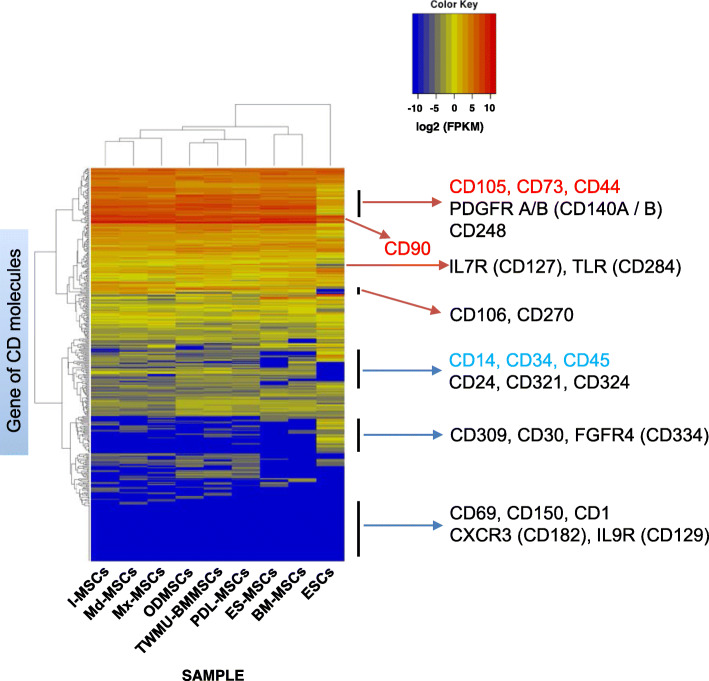
Gene expression profile of CD molecules in each sample. Heatmap showing the degree of expression levels (log2 of FPKM value) of all CD genes. Scaled expression values are color-coded according to the legend on the left. Genes are hierarchically clustered by the similarity of their expression profiles over the set of samples, and the samples are hierarchically clustered by the similarity of expression patterns over their expression profile. The described genes (right side of heatmap) are representative CD molecules. Red letters represent positive markers for MSCs, and blue letters represent negative markers for MSCs
