# Supplementary material for: RNA-sequencing reveals positional memory of multipotent mesenchymal stromal cells from oral and maxillofacial tissue transcriptomes
Source: BMC Genomics. 2020 Jun 22;21:417. doi: 10.1186/s12864-020-06825-2 (PMC7310078; doi:10.1186/s12864-020-06825-2)
Supplement: Supplementary file 1 — Additional file 1: Figure S1. Osteogenic and adipogenic differentiation potential of all MSC samples. Figure S2. Gene expression profile of all HOX genes in I-MSCs, Mx-MSCs, and Md-MSCs. Figure S3. Distinct gene expression patterns between Mfr-MSCs and other cells. [file 12864_2020_6825_MOESM1_ESM.docx]

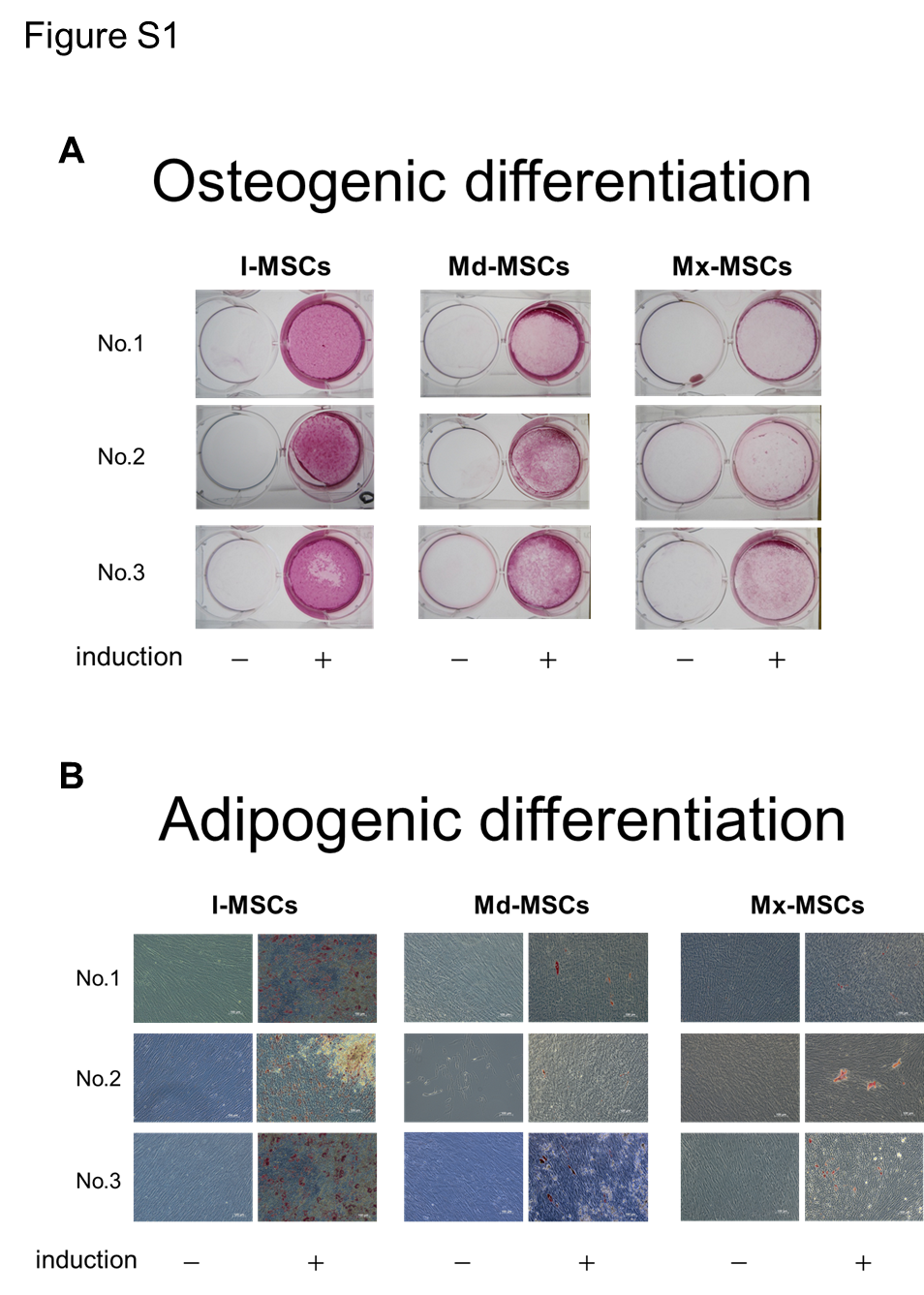


**Figure S1** Osteogenic and adipogenic differentiation potential of all MSC samples. **a** Alizarin red S staining of MSCs cultured in ODM or complete medium for 6 weeks. –, MSCs cultured in complete medium for 6 weeks; +, MSCs cultured in ODM for 6 weeks. **b** Oil red O staining of MSCs cultured in ADM or ODM for 6 weeks. –, MSCs cultured in ODM for 6 weeks; +, MSCs cultured in ODM for 3 weeks, then in ADM for 3 weeks.


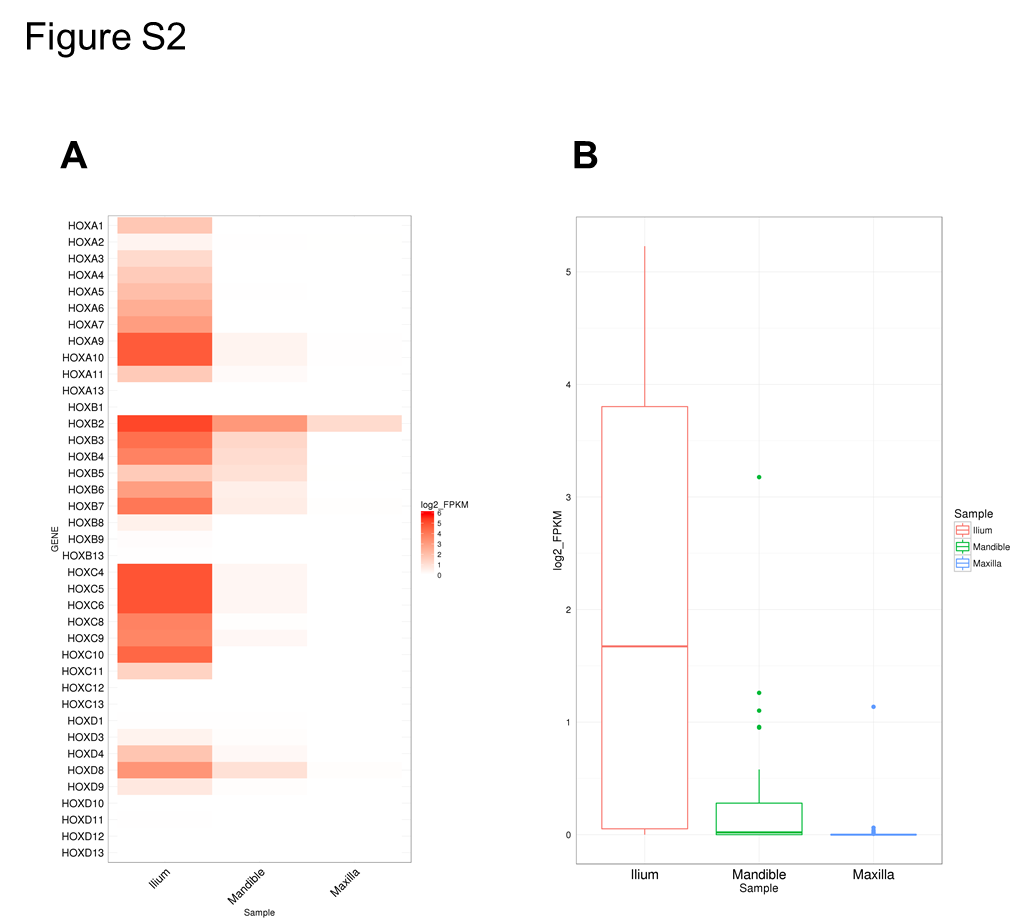


**Figure S2** Gene expression profile of all HOX genes in I-MSCs, Mx-MSCs, and Md-MSCs. **a** Heat map showing the degree of expression levels (*log*2 of FPKM value) of all HOX genes. **b** Box plot showing the distribution of expression levels (*log*2 of FPKM value) of HOX genes (except HOX genes with FPKM = 0 in all samples)


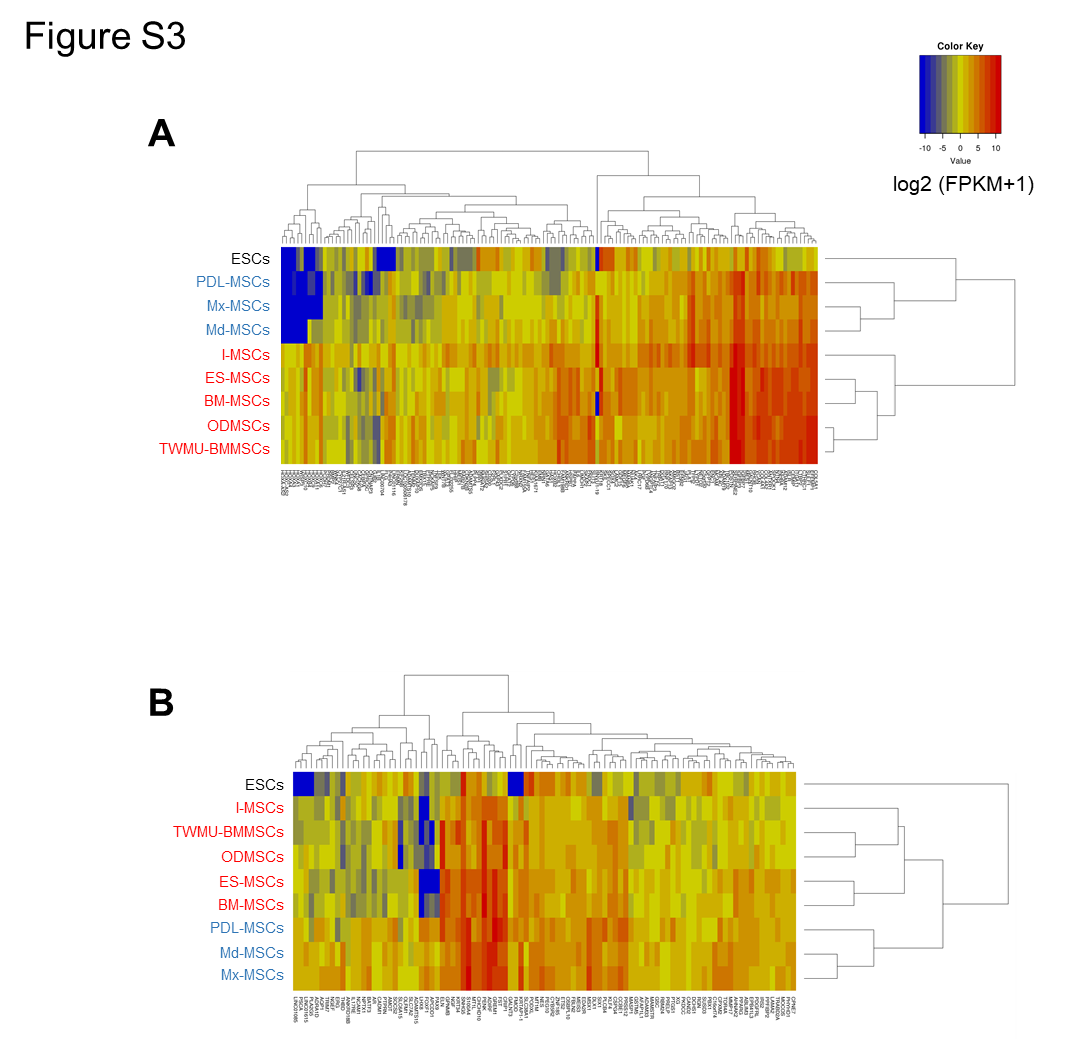


**Figure S3** Distinct gene expression patterns between Mfr-MSCs and other cells. **a, b** Heatmap showing relative expression profiles of U-DEGs (a) and D-DEGs (b). Blue letters represent Mfr-MSCs, and red letters represent other MSCs. Scaled expression values (*log*2 of [FPKM + 1]) are color-coded according to the legend on the right. Genes are hierarchically clustered by the similarity of their expression profiles over the set of samples, and samples are hierarchically clustered by the similarity of expression patterns over their expression profile
