# Supplementary material for: RNA-sequencing reveals positional memory of multipotent mesenchymal stromal cells from oral and maxillofacial tissue transcriptomes
Source: BMC Genomics. 2020 Jun 22;21:417. doi: 10.1186/s12864-020-06825-2 (PMC7310078; doi:10.1186/s12864-020-06825-2)
Supplement: Supplementary file 2 — Additional file 2: Table S1. The information about the subjects. [file 12864_2020_6825_MOESM2_ESM.docx]

**Supplemental Table S1. Isolation of MSCs from different type of bone tissue**

| Gender | Age | Origin |
| --- | --- | --- |
| Male | 33 | Maxilla |
| Male | 26 | Maxilla |
| Female | 8 | Maxilla |
| Male | 22 | Mandible |
| Male | 33 | Mandible |
| Male | 20 | Mandible |
| Male | 9 | Ilium |
| Female | 7 | Ilium |
| Male | 5 | Ilium |
